# High and Low Salt Intake during Pregnancy: Impact on Cardiac and Renal Structure in Newborns

**DOI:** 10.1371/journal.pone.0161598

**Published:** 2016-08-25

**Authors:** Priscila Seravalli, Ivone Braga de Oliveira, Breno Calazans Zago, Isac de Castro, Mariana Matera Veras, Edson Nogueira Alves-Rodrigues, Joel C. Heimann

**Affiliations:** Department of Internal Medicine of the University of São Paulo School of Medicine, São Paulo, Brazil; University Medical Center Utrecht, NETHERLANDS

## Abstract

**Introduction:**

Previous studies from our laboratory demonstrated that dietary salt overload and salt restriction during pregnancy were associated with cardiac and renal structural and/or functional alterations in adult offspring. The present study evaluated renal and cardiac structure and the local renin-angiotensin system in newborns from dams fed high-, normal- or low-salt diets during pregnancy.

**Methods:**

Female Wistar rats were fed low- (LS, 0.15% NaCl), normal- (NS, 1.3% NaCl) or high- (HS, 8% NaCl) salt diets during pregnancy. Kidneys and hearts were collected from newborns (n = 6-8/group) during the first 24 hours after birth to evaluate possible changes in structure using stereology. Protein expression of renin-angiotensin system components was evaluated using an indirect enzyme-linked immunosorbent assay (ELISA).

**Results:**

No differences between groups were observed in total renal volume, volume of renal compartments or number of glomeruli. The transverse diameter of the nuclei of cardiomyocytes was greater in HS than NS males in the left and right ventricles. Protein expression of the AT_1_ receptor was lower in the kidneys of the LS than in those of the NS and HS males but not females. Protein expression of the AT_2_ receptor was lower in the kidneys of the LS males and females than in those of the NS males and females.

**Conclusion:**

High salt intake during pregnancy induced left and right ventricular hypertrophy in male newborns. Salt restriction during pregnancy reduced the expression of renal angiotensin II receptors in newborns.

## Introduction

A previous study from our laboratory demonstrated that adult male Wistar rats fed a high-salt diet and born from dams exposed to salt overload during pregnancy developed left ventricular hypertrophy compared with adult male offspring born from dams fed a normal-salt diet during pregnancy [1]. Maternal high-salt diet during pregnancy may cause cardiac dysfunction in offspring of spontaneously hypertensive rats [2]. Excessive salt intake during pregnancy and lactation is also associated with proteinuria and a low glomerular filtration rate in ninety-day-old Wistar rats [3]. Koleganova et al. found that adult offspring of dams fed high-salt diets and offspring of dams fed low-salt diets during pregnancy and lactation exhibit lower nephron numbers than offspring from dams fed a normal-salt diet during the perinatal period [4]. The feeding of a low-salt diet during the perinatal period is associated with low birth weight and insulin resistance during adulthood [5].

Few studies investigated the effect of the degree of maternal salt intake during gestation and/or lactation on structural and functional alterations in newborns. Whether the described effects of salt overload or restriction during the perinatal period in adult offspring are already present in newborns is not known. Therefore, this study investigated the effects of low- or high-salt intake during pregnancy in Wistar rats on cardiac and renal structures and protein expression of local renin-angiotensin system (RAS) components in newborn offspring.

## Methods

The Committee of Research Projects Evaluation of the University of São Paulo School of Medicine, Brazil previously approved all of the experiments (certificate number 044/14).

### Animals

#### Maternal groups

Eight-week-old female Wistar rats were provided by the institutional animal facility of the University of São Paulo School of Medicine. The animals were housed in a room with controlled temperature from 21 to 22°C and a 12-hour light/dark cycle and given free access to food (NS—1.3% sodium chloride—Nuvilab CR1-Colombo, PR, Brazil) and water. At 12 weeks of age, the females were mated with male Wistar rats that were fed NS. The positive identification of spermatozoids in vaginal smears marked day one of pregnancy. The rats were divided at this time into three groups according to an assigned diet (Harlan Teklad, Madison, WI, USA) until the end of pregnancy: low- (LS, 0.15%), normal- (NS, 1.3%) or high- (HS, 8% NaCl) salt diet according to the protocol described by Rodrigues et al. (2013). Body weight and food consumption were evaluated weekly during pregnancy.

#### Offspring groups

Newborns were weighed, separated by gender and euthanized via decapitation in the first 24 hours after birth. Four to eight offspring from seven or eight dams were evaluated. Kidneys and hearts were collected immediately and weighed. Half of these organs were frozen in liquid nitrogen and stored at -80°C for molecular analyses. The other half were fixed via immersion in 4% paraformaldehyde (PBS, pH 7.4) for 24 h and transferred to 70% ethanol until analyses in stereological studies.

#### Stereological analysis

Kidneys and hearts were routinely processed for paraffin embedding. We embedded the entire kidney and produced sections parallel to its major axis. The entire heart was also embedded, but its major axis was perpendicular to the section plane. Blocks containing kidneys and hearts were exhaustively sectioned at 5 μm using a microtome. Every 60^th^ (kidney) and 70^th^ (heart) slice was collected onto a glass slide until the end of both blocks was reached. We also collected random intervals of 10 pairs of kidney sections (D = 15 μm) to estimate the number of glomeruli. We used a systematic, uniform, random sampling scheme to select sections for analysis [6]. Hematoxylin and eosin were used to stain the sections.

#### Total volumes of the kidney and the heart

We obtained the total volumes of the hearts and the kidneys using the Cavalieri Principle [6].

#### Volume fractions and total volumes of kidney and heart compartments

We used the method of point counting to estimate the volume fraction (Vv comp) of each major compartment of the kidney (cortex, medulla and pelvis) and heart (septum, left ventricle, right ventricle and respective cavities) [7]. We used the same low magnification (2.5×) photomicrographs that were prepared to estimate the organ volume and the same test point system superimposed with the aid of ImageJ software [http://rsb.info.nih.gov/ij/] [8]. We performed differential counts of points falling on the structures of interest and used the following formula: (1) we estimated the Vv comp and multiplied the Vv comp by the total volume (2) of the organ (kidney or heart) to obtain the total volume (V comp) of the compartment.

$$Vv\;comp=\frac{\sum pt\;comp}{\sum pt\;(struct)}$$ (1)

Vcomp=VvcompxVorgan (2)

where ∑pt (comp) is the sum of points falling in a defined compartment in the series of sections analyzed, and ∑pt (struct) is the sum of points falling in the other structures (compartments) of the organ.

#### Total Number of Glomeruli

We determined the glomerular number using the physical dissector method [9]. The same fields of view on a pair of sections were photographed and printed. We used an unbiased counting frame printed on acetate for these counts. These frames were superimposed in both sections, and glomeruli that were sampled by the frame in the first section but not present in the second frame were counted. The count was also performed by comparing the second section of the pair with the first section to double the accuracy. We analyzed six dissectors in each pair of sections in ten pairs of sections. The following formulas (3) (4) were used to obtain the numerical density (Nv) and the total number (N) of glomeruli.

Nv=∑ng/(Vdisx∑pt) (3)

where Σn_G_ is the total number of glomeruli counted in all the dissectors, V_DIS_ is the volume of the dissector (area of the frame x distance between the section pair), and Σpf is the sum of frames with associated points hitting the cortex.

N=NvxVcortex (4)

#### Transverse diameter of the nucleus of cardiomyocytes

Due to the immaturity of the cardiomyocytes and the difficulty of clearly defining the cell borders to assess the transverse diameter, we estimated the diameter of the nucleus, which is a useful indicator of cell hypertrophy [10]. Random fields of view were selected and photographed (40×) for these evaluations. We sampled the nuclei for measurement by superimposing a test system of points. When a point hit a nucleus, it was selected, and two perpendicular measures were performed, one of which was on its major axis. The mean of these two measures was calculated, and at least 100 nuclei were measured for each heart. ImageJ was used for all analyses.

#### ELISA

Whole kidneys and hearts from eight animals from each experimental group of male and female newborns were homogenized (IKA ULTRA-TURRAX T10 Basic, Wilmington, North Carolina, USA) in 500 μL of buffer (50 mM Tris-Cl, 1 mM EDTA + 10% sucrose, pH 7.4), 5 μL of a cocktail of protease inhibitors (Sigma, St. Louis, Missouri, USA) and 100 mM sodium fluoride for each tissue. Homogenates were transferred to Eppendorf tubes and centrifuged at 15,000 × g for 10 minutes. Supernatants were discarded via tube inversion. The pellet was resuspended in 1000 μL of buffer (50 mM Tris-Cl, pH 7.4, 1 mM EDTA) and centrifuged at 15,000 × g for 40 minutes. The supernatants were discarded, and 500 μl buffer (50 mM Tris-Cl, pH 7.4) was added to the pellet and homogenized with a pipette. Protein concentration was measured using a kit (BCA protein assay, Thermo Fisher Scientific, Waltham, MA, USA). Equal amounts of protein (1 μg) were mixed with Tris buffer, pH 7.4, to complete a volume of 100 μl. Each sample was placed in a well of polystyrene ELISA plates (Costar 3590, 96-well flat-bottom plate without a lid, high binding). The plate containing the samples was left in a clean room at room temperature for 24 hours to dry the samples via evaporation. The dried samples were blocked in blocking buffer (1% BSA, 5% sucrose, 0.05% sodium azide, PBS) for 180 minutes, incubated with primary antibodies of angiotensin II receptors AT_1_ and AT_2_. Dilutions of the following antibodies were used: anti-AT_1_ receptor (1:30) (anti-rabbit—Santa Cruz, sc 1173, Dallas, Texas, USA) and anti-AT_2_ receptor (1:30) anti-rabbit (Santa Cruz, sc-9040, Dallas, Texas, USA). The plates were incubated at 4°C overnight.

The plate was washed three times for five minutes with gentle agitation in PBS (200 μl per well) and incubated with 100 μl of a secondary antibody (anti-rabbit IgG, produced in goat and bound to alkaline phosphatase, SIGMA-ALDRICH, A3687, St. Louis, Missouri, USA,) for two hours under gentle agitation. Four washes were performed using PBS buffer for five minutes. After these washes, 100 μl of developing buffer was added (100 mM Tris-base, Sigma 104 phosphatase substrate) and read in an ELISA reader at a wavelength between 400 and 420 nm.

Analyses of variance of one or two factors (one-way or two-way ANOVA) were performed, as appropriate, followed by ad-hoc Tukey, Bonferroni or Fisher tests. Calculations were performed in GraphPad Prism ® version 4.0 software (GraphPad Software, Inc., California, USA) or IBM SPSS version 19.0 (Armonk, New York, USA). The level of statistical significance was set at p<0.05.

## Results

The body weight of dams in the third week of pregnancy was lower (P < 0.05) in the LS group than the HS group. Food consumption was lower (P < 0.05) in the LS group than the NS and HS groups. Birth weight was lower (P < 0.05) in LS than NS and HS males and females. No differences in litter size, sex ratio, cardiac mass or renal mass were observed between the experimental groups (Table 1).

**Table 1:** Effect of low-, normal- and high-salt diet during pregnancy on mothers and offspring.

		LS	n	NS	n	HS	n
Dams							
Body weight—third week of gestation (g)		351±23^#^	8	377±36	7	386±42	8
Food intake (g/dam/day)							
Seventh day of gestation		21±1	7	21±1	7	20±1	7
Fourteenth day of gestation		21±1	7	22±1	7	22±1	7
Twenty first day of gestation		19±1*^#^	7	22±1	7	22±1	7
Neonates							
Litter size		13.6±0.8	8	14±0.6	7	14±0.5	9
	M	6.9±1.0	8	7.6±0.8	7	6.7±0.8	9
	F	6.7±0.6	8	6.4±0.7	7	7.4±0.8	9
Body weight (g)	M	5.4±0.09*^#^	55	6.4±0.07	53	6.4±0.08	60
	F	5.1±0.07*^#^	53	6.0±0.09	45	6.0±0.08	67
Sex ratio	M	6.9±1.0	8	7.6±0.8	7	6.7±0.8	9
	F	6.7±0.6	8	6.4±0.7	7	7.4±0.8	9
Heart mass/ body weight (mg/10 g)	M	59±2	23	63±2	28	60±2	26
	F	0.061±0.002	25	0.062±0.001	24	0.059±0.002	32
Kidney mass/body weight (mg/10 g)	M	94±3	23	99±2	28	92±0.004	26
	F	102±3	25	100±2	24	97±2	32

Data are reported as the means±SEM. M = Males, F = Females, n = number of animals.

*p<0.05 vs. NS

^#^p<0.05 vs. HS.

### Volumes of offspring hearts and compartments

he total cardiac volume was lower in the LS than NS male offspring. There were no differences between the volumes of the compartments: right ventricle, right ventricular cavity, interventricular septum, left ventricular or left ventricular cavity. The total cardiac volume was not different between the female offspring of the experimental groups. The volume fraction of the left LV cavity was lower in the LS group than the HS group, and the interventricular septum volume fraction was greater in the LS group than the HR group (Table 2).

**Table 2:** Heart and compartment volumes in the newborn offspring from dams fed a low- (LS), normal- (NS) or high-salt (HS) diet during pregnancy.

		LS	NS	HS
Heart total volume (mm^3^)	M	2.57±0.14*	3.09±0.12	3.09±0.20
	F	2.36±0.10	2.97±0.37	2.81±0.21
Left ventricle (mm^3^)	M	0.80±0.08	0.91±0.09	0.88±0.06
	F	0.65±0.05	0.72±0.08	0.78±0.08
Left ventricular cavity (mm^3^)	M	0.18±0.03	0.30±0.05	0.26±0.04
	F	0.65±0.05	0.72±0.08	0.78±0.08
Right ventricle (mm^3^)	M	0.68±0.04	0.76±0.05	0.74±0.10
	F	0.71±0.08	1.01±0.13	0.84±0.07
Right ventricle cavity (mm^3^)	M	0.19 ±0.02	0.25±0.02	0.27±0.04
	F	0.22±0.01	0.38±0.09	0.30±0.03
Septum (mm^3^)	M	0.30±0.05	0.34±0.02	0.40±0.03
	F	0.21±0.03	0.15±0.02	0.18±0.03
Left ventricle (%)	M	0.31±0.02	0.29±0.02	0.29±0.02
	F	0.27±0.01	0.25±0.02	0.28±0.01
Left ventricle cavity (%)	M	0.07±0.01	0.10±0.01	0.08±0.01
	F	0.09±0.01^#^	0.10±0.01	0.10±0.01
Right ventricle (%)	M	0.27±0.02	0.25±0.02	0.24±0.02
	F	0.30±0.03	0.34±0.02	0.30±0.01
Right ventricle cavity (%)	M	0.08±0.01	0.08±0.01	0.09±0.01
	F	0.09±0.00	0.12±0.02	0.30±0.01
Septum (%)	M	0.12±0.01	0.11±0.01	0.13±0.00
	F	0.09±0.01*	0.05±0.00	0.06±0.01

Data are reported as the means±SEM. n = 6/group, M = Males, F = Females *p<0.05 vs. NS

^#^p<0.05 vs. HS.

### Volumes of offspring kidneys and compartments and glomeruli number

Total kidney volume and compartment volumes (cortex, medulla and pelvis) were not different between the three experimental groups in newborn males and females. The number of glomeruli was also not different between the experimental groups, but female neonates exhibited a higher number of glomeruli than males in the three experimental groups (Table 3).

**Table 3:** Volumes of whole kidneys and compartments in newborns.

		LS	NS	HS
Kidney total volume (mm^3^)	M	7.04±0.47	7.44±0.22	8.20±0.46
	F	6.73±0.61	5.84±0.44	7.25±0.98
Cortex (mm^3^)	M	4.97±0.34	5.40±0.16	5.86±0.27
	F	4.74±0.48	4.19±0.28	4.99±0.61
Medulla (mm^3^)	M	1.89±0.16	1.87±0.07	2.16±0.16
	F	1.76±0.15	1.51±0.16	2.08±0.36
Pelvis (mm^3^)	M	0.18±0.04	0.17±0.02	0.18±0.06
	F	0.22±0.03	0.14±0.04	0.18±0.04
Cortex (%)	M	0.71±0.01	0.73±0.00	0.72±0.01
	F	0.70±0.01	0.72±0.02	0.70±0.01
Medulla (%)	M	0.27±0.01	0.25±0.01	0.26±0.01
	F	0.26±0.01	0.26±0.02	0.28±0.01
Pelvis (%)	M	0.03±0.01	0.02±0.00	0.02±0.01
	F	0.04±0.01	0.02±0.00	0.03±0.01
Glomeruli number	M	16286±1351	16318±1085	14405±1499
	F	24095±2431*	25770±3096*	24112±2938*

Data are reported as the means±SEM. n = 5–6, M = Males; F = Females.

*p<0.05 females vs. males.

### Protein Expression of AT_1_ and AT_2_ receptors in kidney and heart

**Fig 1:**
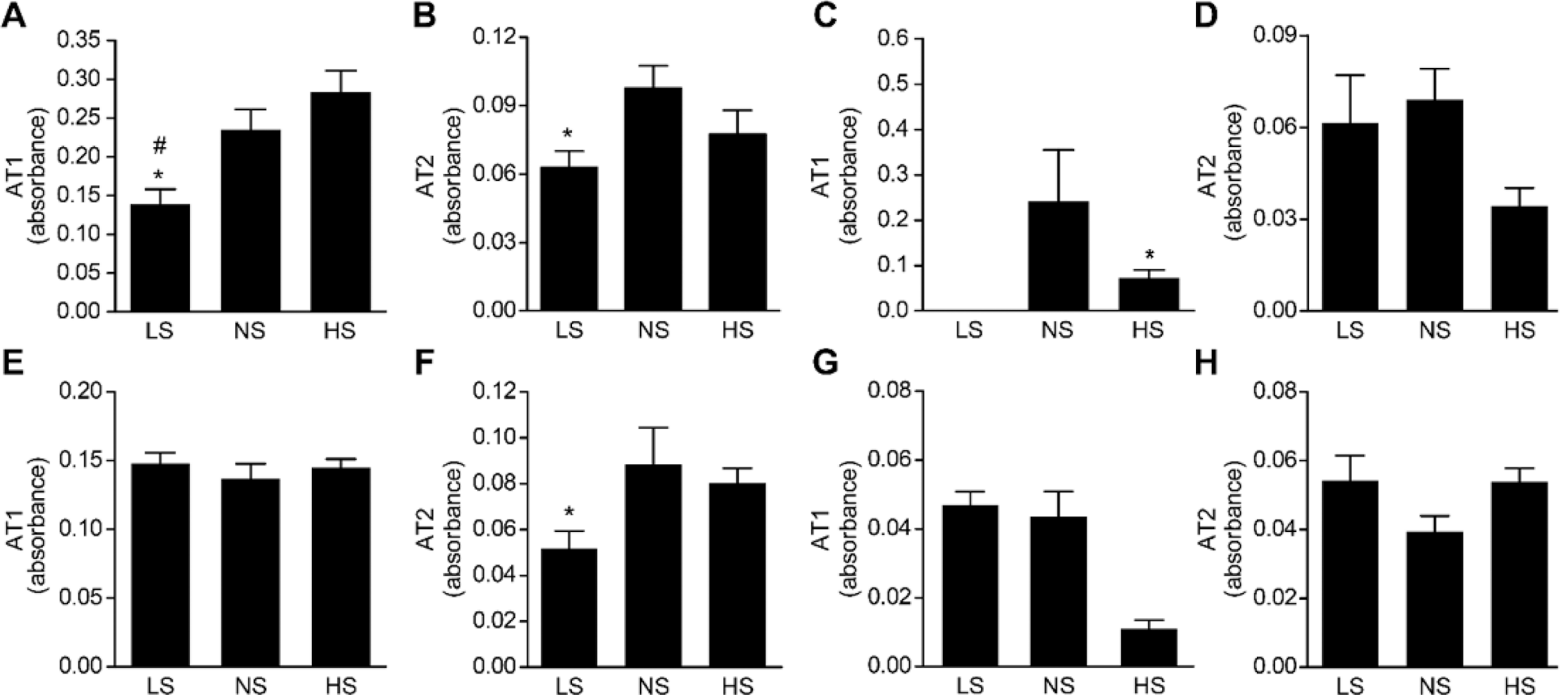
Protein expression in the kidney and heart of newborn offspring. (A) AT_1_ in male kidneys; (B) AT_2_ in male kidneys; (C) AT_1_ in male heart; (D) AT_2_ in male heart; (E) AT_1_ in female kidneys; (F) AT_2_ in female kidneys; (G) AT_1_ in female heart; (H) AT_2_ in female heart. n = 8 per group. Data are presented as the means±SEM. *P<0.05 vs. NS; ^#^ P<0.05 vs. HS.

Protein expression of the AT_1_ receptor in kidneys was lower (p > 0.05) in male newborns of the LS group than the NS and HS groups. AT_1_ receptor protein expression in the kidneys of females did not differ between the experimental groups. However, AT_1_ receptor protein expression in the kidney was higher (p < 0.05) in males than females. AT_2_ receptor protein expression was lower in neonatal males and females in the LS than the NS group. AT_1_ protein expression in the heart was absent in male, but not female, neonates in the LS group. AT_2_ receptor protein expression did not differ between the experimental groups in neonatal males and females (Fig 1).

### Nucleus transverse diameter of cardiomyocytes

**Fig 2:**
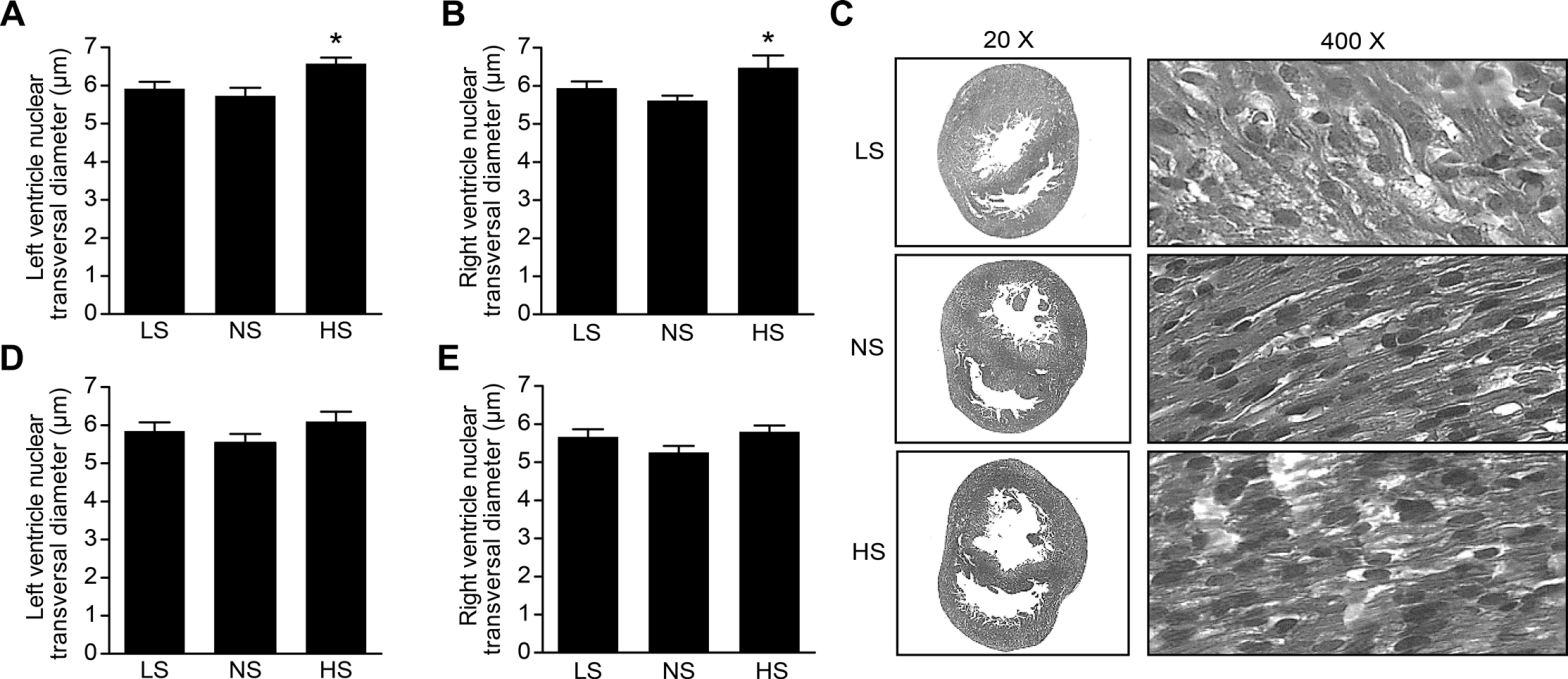
Cardiomyocyte nuclear diameter in neonatal offspring. (A) Left ventricle nuclear diameter (μm) in males. (B) Right ventricle nuclear diameter (μm) in males. (D) Left ventricle nuclear diameter (μm) in females. (E) Right ventricle nuclear diameter (μm) in females. (C) Male cardiac tissue sections stained with HE at low (20 X) and high (400 X) magnification. LS = Newborns of mothers fed a low-salt diet, NS = normal-salt diet, HS = high-salt diet during gestation (n = 5–6/ group). Data are reported as the means±SEM. *P<0.05 vs. NS.

The transverse diameters of the nuclei of cardiomyocytes of the left and right ventricles of male offspring were higher in the HS group than the NS group. No differences were observed in the nucleus transverse diameters of cardiomyocytes between female experimental groups (Fig 2).

## Discussion

The present study revealed some new and relevant findings in neonates in response to dietary salt overload or restriction during gestation. A low-salt intake during pregnancy was associated with lower body weight and food consumption during the third week of gestation and lower birth weight. Cardiomyocyte hypertrophy was observed in male newborns from dams fed a high-salt diet during pregnancy. Lower cardiac volume and absent AT_1_ protein expression was found in male, but not female, newborns from dams fed a low-salt diet. The dietary salt content of the dams during gestation differentially influenced protein expression of the renin-angiotensin system in neonatal males and females.

Undernourishment is an unlikely mechanism to explain the lower maternal body weight and lower offspring birth weight because food intake was lower only during the last week of gestation in salt-restricted dams. Low body weight and low birth weight were also found in previous studies of low caloric or protein intake during pregnancy [11,12,13]. However, a 50% caloric or protein restriction during the entire pregnancy was the most frequently used experimental protocol, and it is different from the present study. Food intake in the present study was only 14% lower in pregnant dams fed a low-salt diet, which is much lower than the 50% reduction used in previous studies [11,12,13]. The association between low birth weight and salt restriction during pregnancy in the present study corroborates previous studies from our laboratory [1,5,14]. No difference was observed in offspring sex ratio due to low or high salt intake during pregnancy in the present study, which is different from the observations of Gray et al. [15] who found a higher ratio of males in response to salt overload during gestation. These conflicting results may be due to protocol differences. Gray et al. studied Sprague-Dawley rats and used a 4% NaCl content. The present study used Wistar rats and an 8% NaCl dietary content in the high-salt pregnant rats. However, the association between salt restriction during pregnancy and lower weight gain and food intake during the third week in rats is also not a consistent finding. Siqueira et al. [14] did observe lower weight gain during the third week of gestation in dams fed low salt diet, but Vidonho et al. [5] reported the opposite results. Because low birth weight was observed in both studies, it is not necessarily a consequence of lower weight gain during pregnancy. Under-nutrition during gestation is associated with a lower number of nephrons, a faster progression of renal disease and increased cardiovascular risk in offspring [16, 17,18]. The occurrence of maternal malnutrition is unlikely in this study because no differences in glomeruli number were found between the experimental groups. Renal mass and volume and cardiac mass were not different between the experimental groups despite the lower birth weight in LS neonates, which suggests that low salt intake during gestation does not interfere with whole body fetal growth and is limited to some organs and/or tissues. Nephrogenesis in rodents is completed at least eight days after birth [19]. Therefore, the absence of differences in the number of mature glomeruli in newborns in the present study may change one week after birth. A study by Koleganova et al. [4] supports this hypothesis. These authors found lower glomeruli number in seven-day-old offspring of dams fed low- or high-salt diets during pregnancy and lactation. Therefore, the different results observed by Koleganova et al. [4] and the present study may be due to differences in the study protocol. Koleganova et al. [4] evaluated Sprague-Dawley rats and counted all glomeruli in seven-day-old offspring. The present study counted only mature glomeruli in one-day-old neonates. Gray et al. [20] also did not find fetal glomeruli number differences at gestation day 20 in response to 4% or 0.26% NaCl dietary content. Further studies are needed to clarify this issue. The higher number of glomeruli in female newborns compared to male newborns independent of the degree of salt intake during pregnancy confirms previous results from our group [21]. Further studies are required to confirm this finding and delineate the mechanism of the gender difference at an early stage of life.

Changes in placental function also influence newborn weight regardless of maternal nutritional status. A previous study from our group [22] demonstrated that placental mass was higher in dams fed a high-salt diet, and the opposite effect was observed in dams fed a low-salt diet. These results indicate that the low birth weight of offspring of dams fed a low-salt diet is likely due to limitations in placental nutrient transport.

A comment about the salt content of the high-salt diet is worthwhile. Eight percent NaCl is a very high salt content in human parameters, and this level may influence rat appetite. However, a previous study from our group demonstrated that rats fed a high-salt diet eat more than animals fed normal or low-salt diets. High salt intake may increase body water content. Tissue dry weight may be measured to confirm or eliminate this effect. This measurement was not performed, but a previous study reported increased body water content in response to high salt intake, which was unlikely due to the absence of differences in hematocrit between the dietary groups [23].

Notably, the present study found that the degree of salt intake during pregnancy influenced cardiac and renal protein expression of angiotensin II receptors in neonates. Lower and absent AT1 receptor protein expression in LS neonates was observed in the kidneys and hearts, respectively, in males but not females. The precise mechanism of these findings requires further study. However, salt restriction or overload in the heart [24,25,26] and kidneys [4] influences local renin-angiotensin system activity. These alterations may have occurred in the dams during pregnancy in the present study and influenced the local RAS in neonates via an unknown mechanism. The lower renal AT_2_ receptor protein expression in male and female offspring of dams fed a low-salt diet during pregnancy is a primary interest. This finding was organ-specific because it was not observed in the heart. Several studies in the last two and a half decades demonstrated that AT1 and AT2 receptors are linked with renal development. The AT2 receptor is expressed during the earlier intrauterine phase, and AT1 is expressed during the first two weeks of the postnatal period [27]. It is possible that the lower AT2 receptor protein expression found in neonatal offspring of dams fed LS during pregnancy is linked to kidney structural or functional alterations. However, no differences in renal mass and volume were detected between the experimental groups. Further studies are needed to confirm this hypothesis.

The present study was the first study to provide evidence of cardiomyocyte hypertrophy in the left and right ventricles in neonatal males, but not females, of mothers exposed to salt overload during pregnancy. This observation was based on the larger diameter of the nuclei of cardiomyocytes in this group. We evaluated the nuclear diameter of cardiomyocytes because of the immaturity in newborns, which makes it difficult to define the limits of the cells. Previous studies validated this hypertrophy assessment method [28]. The present study demonstrated that the myocardial structural alterations in adult offspring of dams fed an HS diet described by Alves-Rodrigues et al. [1] are already present at birth. The finding of cardiomyocyte hypertrophy in one-day-old offspring of dams fed a high-salt diet during pregnancy is not a response to pressure overload because it was also observed in the right ventricle, which is a low-pressure compartment. An association between the described cardiomyocyte hypertrophy and renin-angiotensin system activation cannot be excluded even without an increase in AT1 receptor protein expression. Evidence from the literature indicates that angiotensin II increases ventricular growth and cardiomyocyte hypertrophy in Sprague-Dawley rat embryos [29]. The finding of cardiomyocyte hypertrophy in neonates in response to high salt intake during pregnancy has an important translational impact because changes may be introduced to prenatal nutritional counseling in humans. In contrast, heart volume was lower in LS male, but not female, neonates. One possible explanation for this finding may be the absence of AT_1_ receptor protein expression in the cardiac tissue of male LS neonates because AT_1_ receptor stimulation induces cell proliferation and cardiac growth [30].

The present study revealed a subject that is rarely evaluated in the biomedical field. A gender dimorphism of several parameters was observed in newborns independent of the salt intake of the dams during gestation. This study evaluated rats on the first day after birth, and it is unlikely that the gender dimorphism was due to sexual hormone differences. Additional studies are needed to clarify this phenomenon. Several hypotheses may be explored based on studies from different laboratories: alterations in the JAK/STAT genes [31], differences in adiponectin, energy homeostasis and insulin sensitivity [32], differences in hemodynamic parameters [33] and alterations in placental function [34].

In conclusion, high-salt intake during pregnancy was associated with cardiac hypertrophy in one-day-old male offspring. Further experiments are needed to verify the mechanisms responsible for this association. Renal and cardiac AT_1_ receptor protein expression was lower in newborns from dams fed a low-salt diet during gestation. Therefore, both high- and low-salt intake during gestation influenced cardiac structure and the renin-angiotensin system of newborns.

## Limitations of the study

It would relevant to obtain more details about angiotensin II receptors protein expression, such as the identity of the renal structure that contained lower AT2 protein expression in the LS offspring.

Functional experiments with RAS blockers may clarify the mechanism of the link between RAS and cardiomyocyte hypertrophy.
